# THE MULTIDISCIPLINARY HISTORIES OF ACTIVE AGING IN POLAND

**DOI:** 10.1093/geroni/igac059.2650

**Published:** 2022-12-20

**Authors:** Jessica Robbins-Panko

**Affiliations:** Wayne State University, Detroit, Michigan, United States

## Abstract

In contemporary Poland, Universities of the Third Age are the most visible institutional forms of active aging. These lifelong-learning institutions that are specifically for retirees often cultivate ideals of independence through workshops and classes that teach new, and potentially transformative, skills and hobbies (Kobylarek, 2018). Universities of the Third Age in Poland emerged out of the fields of andragogy, pedagogy, and social work, fields that have regional intellectual roots in the late 19th/early 20th-century presocialist era, and are based on radically different ideals of personhood, relationality, and care than those of the contemporary postsocialist neoliberal era (Robbins, 2021). This paper analyzes 1) historical data from institutional archives of two Universities of the Third Age in Poland, and 2) secondary sources on histories of andragogy, pedagogy, and social work, to create a locally grounded intellectual history of active aging in central and eastern Europe. The Polish case offers an opportunity to think across divergent political-economic eras, in which assumptions about the value of a person to society have shifted. By tracing how the fields of andragogy, pedagogy, and social work have shaped active aging in Poland, this paper finds that 1) dichotomies of East/West, socialist/capitalist, and individual/collective are insufficient to explain the history of contemporary practices of active aging, and 2) intellectual history can reveal complex relations between political-economic change, and ideals and practices of aging. These findings have implications for advancing gerontological theories of 1) active aging in cross-cultural contexts, and 2) how active aging relates to sociopolitical change.

